# Characterization of Lethal Zika Virus Infection in AG129 Mice

**DOI:** 10.1371/journal.pntd.0004682

**Published:** 2016-04-19

**Authors:** Matthew T. Aliota, Elizabeth A. Caine, Emma C. Walker, Katrina E. Larkin, Erwin Camacho, Jorge E. Osorio

**Affiliations:** Department of Pathobiological Sciences, University of Wisconsin-Madison, Madison, Wisconsin, United States of America; University of California, Berkeley, UNITED STATES

## Abstract

**Background:**

Mosquito-borne Zika virus (ZIKV) typically causes a mild and self-limiting illness known as Zika fever, which often is accompanied by maculopapular rash, headache, and myalgia. During the current outbreak in South America, ZIKV infection during pregnancy has been hypothesized to cause microcephaly and other diseases. The detection of ZIKV in fetal brain tissue supports this hypothesis. Because human infections with ZIKV historically have remained sporadic and, until recently, have been limited to small-scale epidemics, neither the disease caused by ZIKV nor the molecular determinants of virulence and/or pathogenicity have been well characterized. Here, we describe a small animal model for wild-type ZIKV of the Asian lineage.

**Methodology/Principal Findings:**

Using mice deficient in interferon α/β and Ɣ receptors (AG129 mice), we report that these animals were highly susceptible to ZIKV infection and disease, succumbing within seven to eight days. Rapid viremic dissemination was observed in visceral organs and brain; but only was associated with severe pathologies in the brain and muscle. Finally, these results were consistent across challenge routes, age of mice, and inoculum doses. These data represent a mouse model for ZIKV that is not dependent on adapting ZIKV to intracerebral passage in mice.

**Conclusions/Significance:**

Foot pad injection of AG129 mice with ZIKV represents a biologically relevant model for studying ZIKV infection and disease development following wild-type virus inoculation without the requirement for adaptation of the virus or intracerebral delivery of the virus. This newly developed Zika disease model can be exploited to identify determinants of ZIKV virulence and reveal molecular mechanisms that control the virus-host interaction, providing a framework for rational design of acute phase therapeutics and for vaccine efficacy testing.

## Author Summary

Zika virus (ZIKV) is an arbovirus that belongs to the family *Flaviridae*. It currently is causing an explosive outbreak of febrile disease in the Americas. In humans, ZIKV infection typically causes a mild and self-limiting illness known as Zika fever, which often is accompanied by maculopapular rash, headache, and myalgia. During the current outbreak, ZIKV infection during pregnancy has been hypothesized to cause microcephaly and other diseases. The detection of ZIKV in fetal brain tissue supports this hypothesis. Our understanding of the molecular mechanisms controlling ZIKV virulence and pathogenesis is limited, with progress greatly impeded by the lack of a small animal model that does not rely on intracerebral inoculation of the virus. Here, we demonstrate that wild-type ZIKV, that is representative of the Asian lineage currently circulating in South America, causes lethal disease in mice deficient in interferon α/β and Ɣ receptors. In these animals, ZIKV causes severe brain pathology, potentially emulating hallmark features of human fetal ZIKV infection. This newly developed Zika disease model can be exploited to identify determinants of ZIKV virulence and reveal molecular mechanisms that control the virus-host interaction, providing a framework for rational design of acute phase therapeutics and for vaccine efficacy testing.

## Introduction

*Zika virus* (ZIKV; *Flaviviridae*, *Flavivirus*) has emerged out of Africa and caused outbreaks of febrile disease in Yap islands of the Federated states of Micronesia [1], French Polynesia [2], and Oceania; and in late 2015 Brazil reported the first known local transmission of ZIKV in the Americas [3]. The current outbreak in the Americas is cause for great concern because ZIKV appears to be spreading nearly uncontrolled with at least 24 countries and territories experiencing autochthonous spread, including Puerto Rico. Eventual local spread in the southern United States seems inevitable. Clinically, infection with ZIKV resembles dengue fever and several other arboviral diseases [4], but has been linked to neurological syndromes and congenital malformation [5,6]. Alarmingly, the rate of microcephaly (small head, reduced brain size, impaired neurocognitive development) in infants born to pregnant women has increased significantly (20-fold in 2015) in areas with high ZIKV incidence in Brazil [7]. There is increasing evidence that ZIKV infection may be causing this and other birth defects [6]; resulting in numerous CDC travel advisories and several countries recommending women delay pregnancy for up to two years. Prior to this, ZIKV existed in relative obscurity with only sporadic human infections until the end of the last century [8]. The virus is believed to have originated in Africa, where it still circulates enzootically among unknown vertebrate hosts (presumably nonhuman primates), and is transmitted by arboreal *Aedes* mosquitoes [9,10]. These cycles lead to sporadic outbreaks of spillover infection in Africa, but most human cases around the globe result from ZIKV emergence into a human-mosquito cycle involving *Aedes aegypti* [11] and/or other urban or peri-urban *Aedes* species, e.g., *Aedes albopictus* and *Aedes hensilli* [1,12,13]. Despite the continued spread of the virus, there remain no effective antiviral therapies or licensed vaccines.

Establishing a small-animal model therefore is an important step in understanding the mechanisms underlying Zika pathogenesis and immunity. Small animal models for ZIKV are lacking. The one exception has involved adapting ZIKV to intracerebral passage in either newborn mice [14,15] or in five- to six-week-old mice [16,17]. In fact, we are not aware of any mouse model that does not rely on delivery of virus via intracranial inoculation. Nonhuman primate models may be sufficient for vaccine and therapeutic testing; however, they are prohibitively expensive for studies aiming to understand ZIKV pathogenesis and vaccinology. Therefore, we sought to develop a murine infection model of ZIKV. Since it recently has been shown that ZIKV is highly sensitive to the antiviral effects of type I and II interferons (IFN) [18] and that type I and II IFNs control replication and dissemination of many viruses, including other flaviviruses [19–23], we hypothesized that IFN-deficient mice would be susceptible to wild-type ZIKV infection. Herein, we describe our efforts to improve upon the existing small animal ZIKV disease models by characterizing lethal ZIKV infection in AG129 mice.

## Methods

### Ethics statement

This study was carried out in strict accordance with recommendations set forth in the National Institutes of Health *Guide for the Care and Use of Laboratory Animals*. All animals and animal facilities were under the control of the School of Veterinary Medicine with oversight from the University of Wisconsin Research Animal Resource Center. The protocol was approved by the University of Wisconsin Animal Care and Use Committee (Approval #V01327).

### Cells and viruses

African Green Monkey kidney cells (Vero; ATCC #CCL-81) were grown in Dulbecco’s modified Eagle medium (DMEM) supplemented with 10% fetal bovine serum (FBS; Hyclone, Logan, UT), 2 mM L-glutamine, 1.5 g/l sodium bicarbonate, 100 U/ml of penicillin, 100 μg/ml of streptomycin, and incubated at 37°C in 5% CO_2_. *Aedes albopictus* mosquito cells, (C6/36; ATCC #CRL-1660) were maintained in MEM supplemented with 10% FBS, 2 mM L-glutamine, 1.5 g/l sodium bicarbonate, 0.1 mM non-essential amino acids, 100 U/ml of penicillin, 100 μg/ml of streptomycin, and incubated at 28°C in 5% CO_2_. ZIKV strain H/PF/2013 (GenBank:KJ776791), originally isolated from a 51-year-old female in France returning from French Polynesia with a single round of amplification on Vero cells, was obtained from Xavier de Lamballerie (European Virus Archive, Marseille France). Virus stocks were prepared by inoculation onto a confluent monolayer of C6/36 mosquito cells.

### Plaque assay

All ZIKV screens and titrations for virus quantification were completed by plaque assay on Vero cell cultures. Duplicate wells were infected with 0.1 ml aliquots from serial 10-fold dilutions in growth media and virus was adsorbed for one hour. Following incubation, the inoculum was removed, and monolayers were overlaid with 3 ml containing a 1:1 mixture of 1.2% oxoid agar and 2X DMEM (Gibco, Carlsbad, CA) with 10% (vol/vol) FBS and 2% (vol/vol) penicillin/streptomycin. Cells were incubated at 37°C in 5% CO_2_ for four days for plaque development. Cell monolayers then were stained with 3 ml of overlay containing a 1:1 mixture of 1.2% oxoid agar and 2X DMEM with 2% (vol/vol) FBS, 2% (vol/vol) penicillin/streptomycin, and 0.33% neutral red (Gibco). Cells were incubated overnight at 37°C and plaques were counted.

### Mice

Mice of the 129/Sv background deficient in alpha/beta interferon (IFN-α/β) and IFN-Ɣ receptors (AG129 mice) were obtained from B&K Universal Limited (Hull, England) and were bred in the pathogen-free animal facilities of the University of Wisconsin-Madison School of Veterinary Medicine. Groups of three- to four-week-old or eight-week-old mixed sex mice were used for all experiments.

### Mortality and pathogenesis studies

For morbidity/mortality studies with eight-week-old mice, animals were infected in both hind foot pads with 1x10^5^ plaque forming units (PFU) of ZIKV in 100 μl (50 μl/foot pad) of animal diluent (AD: 1% heat-inactivated FBS in Dulbecco’s PBS). For three- to four-week-old mice, animals were infected in both hind foot pads with 1x10^5^, 1x10^4^, 1x10^3^, 1x10^2^, 10, or 1 PFU of ZIKV in 100 μl (50 μl/foot pad) AD; or infected intraperitoneally (i.p) with 2x10^5^ PFU of ZIKV in 200 μl of AD. Mock-infected mice received AD alone to serve as experimental controls. Following infection, mice were monitored twice daily for the duration of the study. Mice that were moribund or that lost greater than 20% of starting weight were humanely euthanized. Sub-mandibular blood draws were performed and serum was collected to verify viremia. Average survival time (AST), percent mortality, and weight changes were calculated.

### Mouse necropsy

Following infection with 10^5^ PFU ZIKV, a group of three mice were sacrificed at seven days post infection (PI) for young mice and a group of four mice were sacrificed at 8 days PI for adult mice. Organ samples were collected in pre-weighed tubes. To determine viral distribution and tissue viral loads, tissues were homogenized using a mixer mill and RNA was extracted from 100 μl of tissue homogenate using the Direct-zol RNA MiniPrep kit (Zymo Research, Irvine, CA). Viral RNA was quantified by qRT-PCR using the primers and probe designed by Lanciotti et al. [24]. The RT-PCR was performed using the iTaq Universal One-Step RT-qPCR kit (BioRad, Hercules, CA) on an iCycler instrument (BioRad, Hercules, CA). Primers and probe were used at final concentrations of 600 nm and 100 nm respectively. Cycling conditions were as follows: 37°C for 15 min, 50°C for 30 min and 95°C for 2 min, followed by 50 cycles of 95°C for 15 sec and 60°C for 1 min. Virus concentration was determined by interpolation onto an internal standard curve made up of a 7-point dilution series of in vitro transcribed RNA.

### Histology

At the time of necropsy, liver, spleen, brain, kidney, intestine, heart, skeletal muscle and lung from four mice for biological replicate one and five mice for biological replicate two were harvested and immediately fixed for 16–24 hours in 10% paraformaldehyde. Tissues for paraffin embedding were submitted to the Histology Laboratory at the School of Veterinary Medicine at the University of Wisconsin-Madison, where they were processed and sectioned before staining with Hematoxylin and Eosin (H&E). Sections were viewed from ZIKV-infected and mock-infected samples and slides were analyzed for histopathological changes. H&E-stained sections were viewed by light microscopy.

### Statistical analyses

All data were analyzed with GraphPad Prism software (Graphpad Software, Inc). For survival analysis, Kaplan-Meier survival curves were analyzed by the log-rank test. An unpaired Student's t-test was used to determine significant differences in virus titers.

## Results and Discussion

### ZIKV infection is fatal for AG129 mice

**Fig 1:**
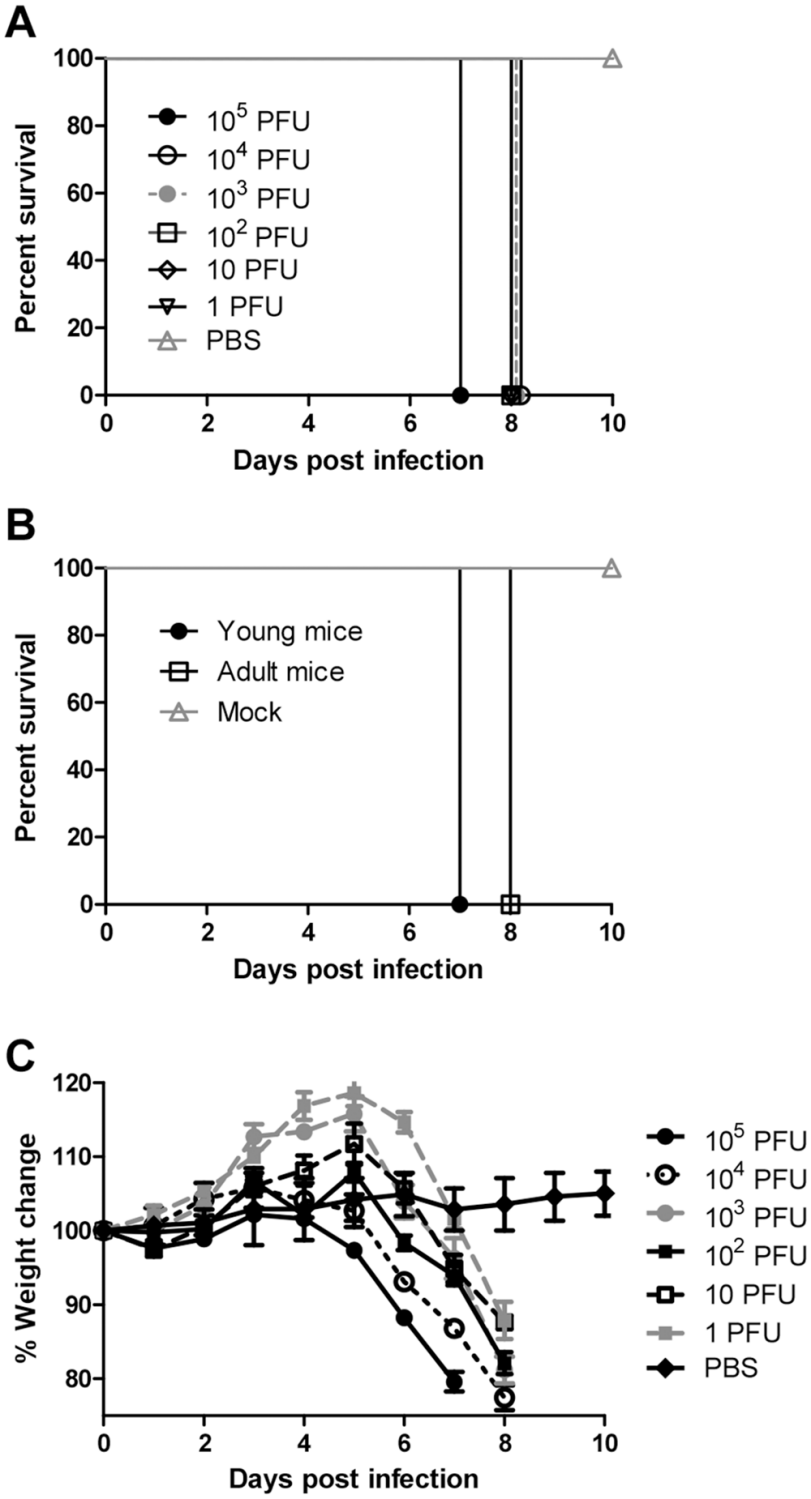
ZIKV causes mortality and morbidity in AG129 mice. Kaplan-Meier curves illustrate the susceptibility of AG129 mice to ZIKV. Young mice were inoculated f.p. with several doses (n = 3 for doses 10^5^−10^3^ PFU and n = 6 for doses 10 and 1 PFU; 10^2^ PFU was done as two separate independent replicates with n = 3 for each) of ZIKV (**A**). All mice challenged with 10^4^−1 PFU succumbed 8 d PI. Young (n = 4) and adult mice (n = 6) were inoculated f.p. with 10^5^ PFU of ZIKV (**B**). Mice were monitored until day 10 PI. Changes in weight were calculated daily for ZIKV- and mock-infected mice (**C**). Error bars represent standard error of the mean.

In initial studies, AG129 mice were tested for susceptibility to infection with ZIKV, which was originally isolated from a traveler that visited French Polynesia in 2013. Both intraperitoneal inoculation (i.p., n = 5) and foot pad inoculation (f.p., n = 4) of three- to four-week-old AG129 mice (young mice) with 10^5^ PFU of ZIKV caused rapidly fatal infection, i.e., both groups were humanely euthanized on day 7 when all mice appeared moribund. In subsequent testing with young mice (n = 3 mice for doses 10^5^−10^3^ PFU and n = 6 for doses 10^2^−1 PFU), 100% mortality was observed for all doses ranging from 10^5^ to 1 PFU (Fig 1A). Likewise, 10^5^ PFU of ZIKV also resulted in 100% mortality in eight-week-old mice (adult mice, n = 6, Fig 1B). Regardless of dose or age, AG129 mice exhibited signs of illness by four to five days PI, including weight loss, lethargy, and hunched posture. Interestingly, none of the mice that succumbed to infection developed signs of paralysis during the entire observation period. Still, animals deteriorated rapidly, becoming immobile and weak, and were typically euthanized seven to eight days PI. As a measurement of mouse morbidity, weight change was monitored daily during acute infection. In contrast to uninfected age-matched controls, which did not exhibit weight loss, ZIKV-infected mice lost weight starting on day five PI (Fig 1C), at approximately the same time as clinical signs were visually noted. Mouse weight decreased to 80% or lower even for mice infected with the lowest dose inoculum of 1 PFU. Weight loss data were consistent with survival data.

**Fig 2:**
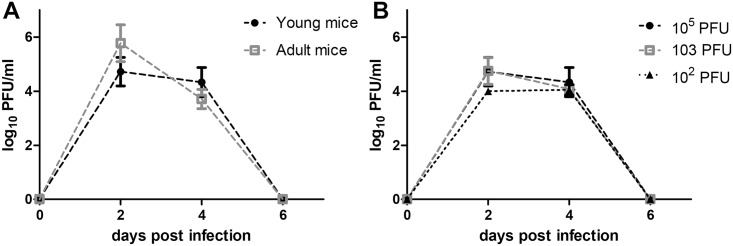
Mice have high serum viremia early during infection. (**A**) Viral titers in the blood of young vs. adult mice receiving 10^5^ PFU of ZIKV at days two, four, and six PI. (**B**) Viral titers in the blood of mice receiving different doses of ZIKV at days two, four, and six PI. Symbols indicate the mean value between individual mice (n = 3 for young mice and n = 6 for adult mice) ± standard deviation.

**Fig 3:**
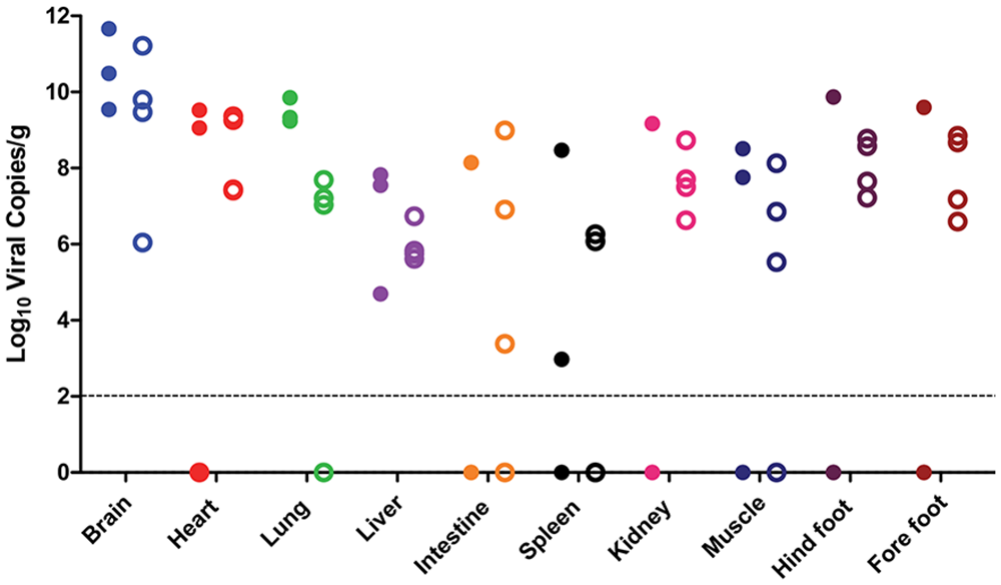
Mice have high tissue viral loads 7 days post infection. Mice were infected with 10^5^ PFU of ZIKV, euthanized at day seven (young) or day eight (adult), and tissue viral loads were determined by qRT-PCR as described in the text. Closed symbols indicate young mice (n = 3), open symbols indicate adult mice (n = 4), and the dotted line represents the limit of detection for the assay.

In order to characterize ZIKV-induced disease further, we measured viral titer in serum from young AG129 mice infected with 10^5^, 10^3^, and 10^2^ PFU of ZIKV and adult AG129 mice infected with 10^5^ PFU (Fig 2). In young mice, viral titer in the serum was observed to peak on day two in all animals infected with ZIKV. No significant differences in viral titers were observed between inoculum doses (p>0.05). In adult mice, viral titer in the serum also peaked on day two and there was no significant difference in viral titers between young and old mice (p>0.05). We also attempted to determine systemic spread of the virus using qRT-PCR. In the absence of type I and II IFN responses, infection with ZIKV led to rapid viral dissemination, most likely by infecting and commandeering migratory dendritic cells and/or macrophages, as has been reported for ZIKV [18] and other flaviviruses [25,26]. Viral loads were high in all tissues and were comparable between young and old mice receiving 10^5^ PFU of ZIKV (Fig 3). The highest viral loads were observed in brains from young mice (e.g., one mouse had 11.69 log_10_ viral copies/g in the brain). Because the animals were not perfused with phosphate-buffered saline prior to tissue collection, some virus detection may be from blood; however, no detectable serum viremia was observed in mice after six days PI.

### ZIKV causes severe pathology in the brain but not in the visceral organs of AG129 mice

**Fig 4:**
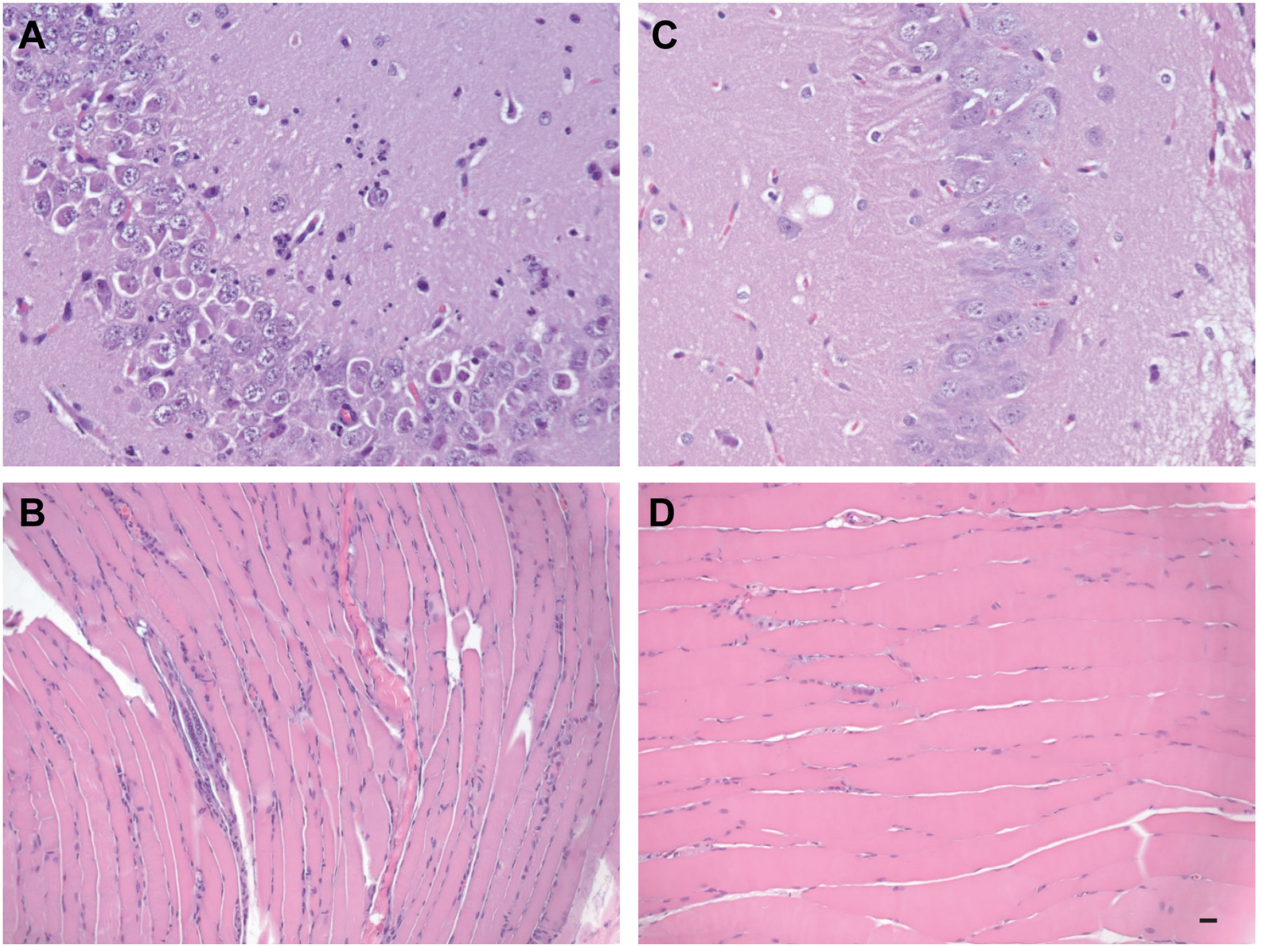
Comparative histological imaging of skeletal muscle and brain after mock infection and infection with ZIKV. Musculature from the posterior rear limb of a ZIKV-infected mouse revealing nuclear rowing as well as degenerate muscle fibers and infiltrating inflammatory cells (**A**). Hippocampal section from a ZIKV-infected mouse revealing neutrophilic infiltration (**B**). Musculature from the same site in a mock-infected mouse (**C**) A section of hippocampus in the brain of mock-infected mouse (**D**). Scale bar, 20 μm. Data are representative of two independent experiments (n = 4 and 5).

Because ZIKV has been associated with microcephaly and recently it was confirmed that ZIKV was present in fetal brain tissue [6], we undertook a comparative histological analysis of brain and other tissues from ZIKV-infected and mock-infected AG129 mice, specifically surveying for obvious morphological changes associated with ZIKV infection. Surprisingly, examination of hematoxylin and eosin-stained semi-thin sections did not reveal any obvious tissue damage associated with ZIKV infection in the majority of tissues examined (e.g., heart, liver, spleen, intestine, kidney, and lung) and looked similar to mock-infected controls. However, examination of the musculature from the posterior rear limb of a ZIKV-infected mouse revealed multi-focal myofiber degeneration and necrosis with inflammatory cell infiltration, nuclear rowing, and attempted regeneration (Fig 4A).

**Fig 5:**
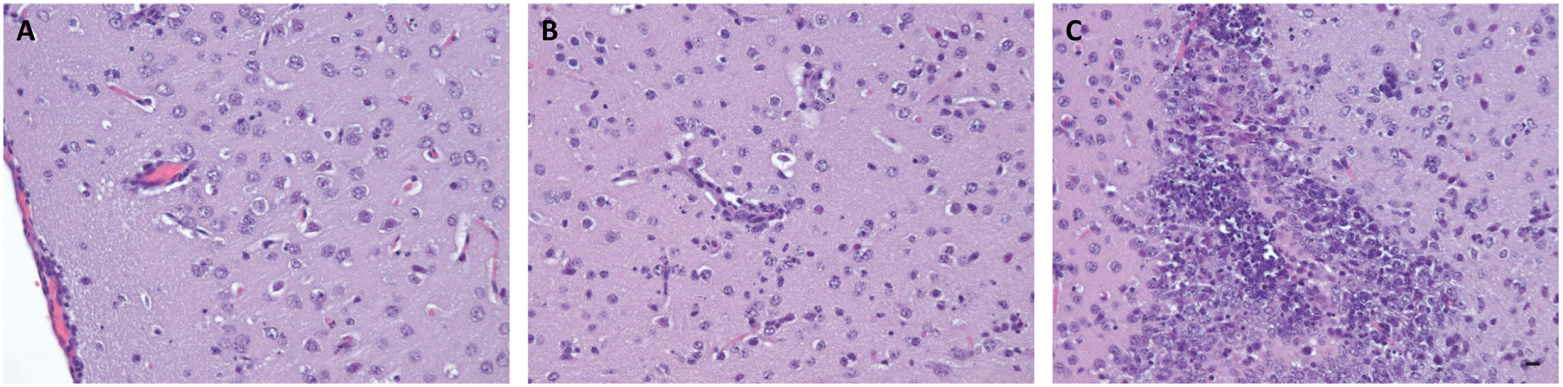
Brain histopathology after ZIKV infection. Cortical tissue revealing meningeal infiltration by a mixture of neutrophils and mononuclear cells. A small amount of a similar infiltrate is seen surrounding an adjacent vessel (**A**). Cerebral neuropil section will cellular pyknosis, scattered neutrophils, and perivascular neutrophilic infiltration (**B**). Apparent necrosis and neutrophil invasion of primordial germ cell region (**C**). Scale bar, 20 μm. Data are representative of two independent experiments (n = 4 and 5).

Examination of the brain, revealed significant histopathology associated with ZIKV infection. The most prominent histopathological feature included neutrophil infiltration of the hippocampus, which at times was associated with degenerate neurons and glia. A prominent linear focus of neutrophil invasion adjacent to the choroid plexus also was observed (Fig 4B), and cortical tissue from the brain showed meningeal infiltration by a mixture of neutrophils and mononuclear cells (Fig 5A). A small amount of a similar infiltrate also was observed surrounding an adjacent small vessel in the cortical tissue. Within the neuropil there were neutrophils and foci of necrotic cellular debris that may have been neurons or glia. This may be indicative of viral replication, because it has been previously demonstrated that ZIKV replicates in both neurons and astroglial cells [17] but confirmation will require further studies/confirmation. In addition, microscopic examination of a section of cerebral neuropil (Fig 5B) revealed cellular pyknosis, scattered neutrophils, and perivascular neutrophilic infiltration. Finally, there was apparent necrosis and neutrophilic invasion of primordial germ cell regions (Fig 5C). In sum, ZIKV caused severe brain pathology in AG129 mice, potentially emulating hallmark features of human fetal ZIKV infection. At the very least, these data warrant further exploration into the feasibility of this infection model in relationship to human disease.

ZIKV was first isolated from a sentinel rhesus monkey in Uganda in 1947 [27], and 20 years later isolated from humans in Nigeria. Since then, the virus has spread to other regions of the world, and now ZIKV has become an emerging global health problem due to recent outbreaks in Micronesia, French Polynesia, Cook Island, Easter Island, and presently the Americas [28]. However, other than its phylogenetic relationship to other mosquito-borne flaviviruses, with few exceptions [e.g., 18], no information is known about molecular mechanisms controlling ZIKV virulence and pathogenesis. Progress with understanding ZIKV pathogenesis has been lacking for myriad reasons, primary among them is the absence of a small animal model that does not rely on intracerebral inoculation of the virus. Similar to what has been demonstrated for DENV in wildtype mice, we postulate that ZIKV proteins likely cannot bind and degrade the homologs for STING [29,30] and STAT-2 [31]; therefore, ZIKV-infected wildtype mice induce efficient IFN-α/β responses that prevent ZIKV replication. Here, we report that ZIKV is capable of causing morbidity and mortality in mice lacking IFN-α/β and IFN-Ɣ receptors. Although, AG129 mice may not perfectly model ZIKV infection of humans, we believe they will be valuable for studying the virulence/attenuation of ZIKV and developing hypotheses to be tested in focused non-human primate studies. In fact, AG129 mice have been useful to study dengue because they experience a vascular leak-like syndrome with features reminiscent of severe dengue disease in humans (e.g., high viral load and soluble NS1 levels, low platelet count, high TNF-α, IL-6, and IL-10 levels) and display DENV tropism similar to what has been observed in humans [32–35]. Finally, they also will be useful for testing potential vaccines and antiviral compounds against ZIKV, because even though they lack a functional interferon response, AG129 mice do develop normal humoral and cellular T cell responses [36–38]. AG129 have been used for numerous vaccine, antiviral, and supportive therapy studies [e.g., 38,39–41]. While improving animal models for ZIKV will be a major challenge moving forward, this model can be used for a number of applications, as long as the specific characteristics of the model are kept in mind and considered in the context of the questions being asked.
