# Correction: Characterization of Lethal Zika Virus Infection in AG129 Mice

**DOI:** 10.1371/journal.pntd.0004750

**Published:** 2016-05-23

**Authors:** Matthew T. Aliota, Elizabeth A. Caine, Emma C. Walker, Katrina E. Larkin, Erwin Camacho, Jorge E. Osorio

The panels in Fig 4 are switched. The image that appears as part A should appear as part B, and the image that appears for part B should appear as part A. The image that appears as part C should appear as part D, and the image that appears for part D should appear as part C.

**Fig 4:**
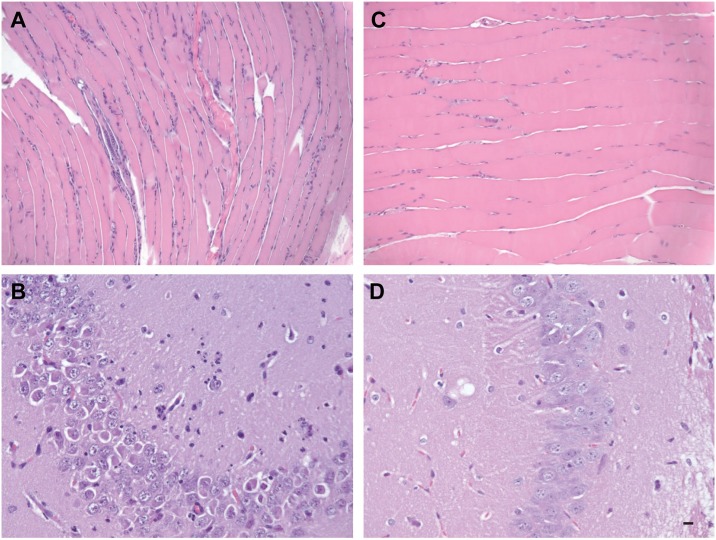
Comparative histological imaging of skeletal muscle and brain after mock infection and infection with ZIKV. Musculature from the posterior rear limb of a ZIKV-infected mouse revealing nuclear rowing as well as degenerate muscle fibers and infiltrating inflammatory cells (**A**). Hippocampal section from a ZIKV-infected mouse revealing neutrophilic infiltration (**B**). Musculature from the same site in a mock-infected mouse (**C**) A section of hippocampus in the brain of mock-infected mouse (**D**). Scale bar, 20 μm. Data are representative of two independent experiments (n = 4 and 5).

## References

[1] Aliota MT, Caine EA, Walker EC, Larkin KE, Camacho E, Osorio JE (2016) Characterization of Lethal Zika Virus Infection in AG129 Mice. PLoS Negl Trop Dis 10(4): e0004682 doi: 10.1371/journal.pntd.0004682 27093158 10.1371/journal.pntd.0004682 PMC4836712

